# Ameliorative Effects of Arctigenin on Pulmonary Fibrosis Induced by Bleomycin via the Antioxidant Activity

**DOI:** 10.1155/2022/3541731

**Published:** 2022-07-05

**Authors:** Yueshang Wang, Xinpeng Li, Shiwen Pu, Xiao Wang, Lanping Guo, Lisheng Zhang, Zhen Wang

**Affiliations:** ^1^College of Animal Science & Technology, College of Animal Veterinary Medicine, Huazhong Agricultural University, Wuhan, 430070 Hubei, China; ^2^College of Pharmacy, Linyi University, Linyi, 276000 Shandong, China; ^3^School of Pharmaceutical Sciences and Key Laboratory for Applied Technology of Sophisticated Analytical Instruments of Shandong Province, Shandong Analysis and Test Center, Qilu University of Technology (Shandong Academy of Sciences), Jinan 250014, China; ^4^State Key Laboratory Breeding Base of Dao-di Herbs, National Resource Center for Chinese Materia Medica, China Academy of Chinese Medical Sciences, Beijing 100700, China

## Abstract

In this study, we evaluated the in vivo effect of arctigenin (ATG) on bleomycin-induced pulmonary fibrosis in mice and assessed the role of antioxidant activity. Hematoxylin and eosin (H&E) staining, the results of Masson's trichrome, and Sirius red staining showed that bleomycin induced obvious pathological changes and collagen deposition in the lung tissue of mice, which were effectively inhibited by ATG. Specifically, based on immunohistochemistry and western blot results, ATG inhibited the expression of fibrosis markers, such as collagen, fibronectin, and *α*-SMA. Moreover, ATG regulated reactive oxygen species (ROS), superoxide dismutase (SOD), malondialdehyde (MDA), and glutathione (GSH) in the lung tissue of pulmonary fibrosis mice and reduced the pressure of oxidative stress. ATG also regulated the TGF-*β*-induced expression of p-Akt, confirming that ATG can inhibit fibrosis through antioxidant activity modulation.

## 1. Introduction

Pulmonary fibrosis (PF) is a common chronic, progressive, and irreversible interstitial lung disease [1]. Pathologically, it is characterized by interstitial cell proliferation and extracellular matrix (ECM) deposition [2, 3]. Once diagnosed, the 5-year average survival rate is only 20%–30%, which is even lower than that of some cancers [4]. At present, the treatment of most interstitial lung diseases is limited, and few drugs are available. Globally, pirfenidone and nintedanib are the leading drugs for the treatment of PF [5]. Although both have been clinically verified to effectively inhibit PF, they have potential side effects, such as gastrointestinal reaction, liver injury, and bleeding, and their efficacy in a variety of fibrotic lung diseases is unclear [6]. PF is a public health problem, and the scarcity of safe and effective drugs seriously limits its treatment options [7]. Therefore, it is urgent to develop safe and effective drugs for PF [8].

In recent years, numerous studies have found that oxidative stress (OS) is closely related to the pathogenesis of PF and plays an important role in its occurrence and development [9, 10]. In 1987, Cantin et al. first published a research report on the relationship between OS and PF [11]. The results showed a significant increase in the ability of inflammatory cells in the bronchoalveolar fluid of patients with PF to release hydrogen peroxide, while myeloperoxidase (MPO) was also detected in the alveolar lavage fluid of patients with PF. The study suggested a correlation between the degree of PF and the level of OS [12]. Several studies have shown that the OS level plays an important role in the process of PF. The mechanism of OS-induced PF mainly involves the injury and necrosis of alveolar epithelial cells (AECs) [13]. The continuous injury and change of AECs are characteristics of PF. Under normal conditions, lung epithelial cells have antioxidant effects, and their stimulation by reactive oxygen species (ROS) can trigger epithelial cell apoptosis to avoid an inflammatory reaction caused by cell necrosis [14]. However, the high concentration of ROS causes the oxidation capacity of epithelial cells to exceed the antioxidant capacity, thereby leading to injury and necrosis, and finally to PF. ROS can induce AEC apoptosis by directly activating death receptors and can also stimulate excessive apoptosis of AECs by activating the mitochondrial apoptosis pathway and the endoplasmic reticulum stress apoptosis pathway. This process leads to an imbalance between matrix metalloproteinases and antiproteases (MMPs/TIMPs) in the lung tissue. Moreover, ROS can regulate TGF-*β* and other cytokines to promote epithelial–mesenchymal transformation (EMT) [15]. These findings suggest that OS plays a key role in the occurrence and development of PF. Indeed, OS can effectively inhibit the process of PF by regulating the oxidation/antioxidant imbalance in the lung tissue [16, 17]. This provides a theoretical basis for finding new potential therapeutic drugs.

*Great Burdock Achene* is the dry and mature fruit of *Arctium lappa*, a biennial herb in the family Compositae. Modern pharmacological studies have shown that arctigenin (ATG), the main component of *Arctium lappa*, has anti-inflammatory, antiviral, and antitumor activities [18, 19]. Previous studies have confirmed that ATG can prevent renal fibrosis by enhancing the active form of antioxidant superoxide dismutase and by inhibiting TGF-*β* expression in rats, which suggests that ATG has good antioxidant activity [20]. Accordingly, we examined the antioxidant activity of ATG to inhibit bleomycin-induced PF in mice by observing the collagen deposition in the lungs.

## 2. Materials and Methods

### 2.1. Reagents

Bleomycin hydrochloride and nintedanib were purchased from Hanhui Pharmaceutical Co., Ltd. (Hangzhou, China) and Boehringer Ingelheim Pharmaceutical Co., Ltd. (Ingelheim, Germany), and arctigenin was purchased from Shanghai Yuanye Bio-Technology Co., Ltd (≥98%, HPLC). All of the other chemicals and reagents were obtained from general commercial sources and were used without prior treatment unless otherwise specified. Antibodies against collagen, fibronectin, *α*-SMA, and TGF-*β* were produced by Affbiotech (Cincinnati, OH, USA); when used in western blot or immunohistochemistry (IHC), they were diluted 1000 or 100 times, respectively, using a primary anti-diluent (Beyotime, Shanghai, China). Antibodies to Nrf-2, HO-1, and NQO1 were produced by ABclonal (Woburn, MA, USA). Secondary antibodies, including biotin-conjugated AffiniPure goat anti-mouse IgG (H + L) and biotin-conjugated AffiniPure goat anti-rabbit IgG (H + L), were provided by Solarbio (Beijing, China). Hydroxyproline and 8-iso-prostaglandin-F2*α* enzyme-linked immunosorbent assay (ELISA) kits were obtained from Bioswamp (Wuhan, China). Malondialdehyde (MDA), glutathione (GSH), and superoxide dismutase (SOD) kits were produced by Solarbio.

### 2.2. Preparation of Different Concentrations of ATG

ATG is insoluble in water but can be dissolved in dimethyl sulfoxide (DMSO). When preparing solutions, a mixture of 5% DMSO and 95% water is required. Specifically, taking the medium dose (40 mg/kg/day) as an example, 160 mg of ATG was dissolved in 2 mL DMSO before adding 38 mL normal saline. A total of 200 *μ*L of the resulting solution was used for injection.

### 2.3. Animals

All of the experiments were performed using male C57BL/6 mice (22 ± 2 g, SPFII Certificate) purchased from Chu Han Biotechnology Co., Ltd. (Jinan, China). The experimental procedures performed in this study were previously approved by the Animal Ethics Committee of the School of Pharmacy at Linyi University. The experiments were also performed in accordance with the ARRIVE guidelines (the ARRIVE guidelines 2.0: updated guidelines for reporting animal research) [21]. All of the methods were performed in accordance with the relevant guidelines and regulations. All efforts were made to minimize the suffering of the animals.

### 2.4. In Vivo Experimental Model and Grouping

Male C57BL/6 mice were randomly divided into six groups, including sham (Sham), bleomycin-induced (3.5 mg/kg) PF (PF), bleomycin-induced PF + nintedanib (Ninb), bleomycin-induced PF + low-dose ATG (20 mg/kg, L-ATG), bleomycin-induced PF + medium-dose ATG (40 mg/kg, M-ATG), and bleomycin-induced PF + high-dose ATG (80 mg/kg, H-ATG) groups, with 10 mice in each group. All of the mice were subjected to 12 : 12 h light/dark cycles for 1 week before experimentation, and all mice were given the same diet throughout the experiment. In the second week, 3.5 mg of bleomycin was dissolved in 1 mL of normal saline, and the injection volume was 1 mL/kg/time [22]. The skin, muscles, and cervical part of the trachea were cut in the Sham group. In the other groups, the skin, muscle, and cervical parts of the trachea were cut, and bleomycin was injected into the trachea. Bleomycin was injected in all of the groups, except for the Sham group, in which saline was injected instead. The mice received ATG by intraperitoneal injection for 28 days [23, 24], whereas the Sham and PF mice were given saline by intraperitoneal injection.

### 2.5. Assessment of Lung Function

After 28 days, the mice were anesthetized with phenytoin sodium (30 mg/kg) and sacrificed. Bilateral lavage was performed on the lungs of the mice. The neck area was dissected, and the trachea was exposed for intubation. The phosphate-buffered saline (PBS) lavage volume was increased to 0.8 mL, and after three lavages, the lavage solution was collected. The recovered alveolar lavage fluid was centrifuged at 1500 r/min for 10 min at 4°C, and the supernatant was recovered and stored at −20°C for ELISA detection.

To test the wet-to-dry ratio of the lung tissue, we took 0.1 g of the lung tissue, weighed it with an analytical balance, and recorded the wet weight. Next, we dried the tissue in an oven at 70°C for 72 h to a constant weight, before weighing again and recording the dry weight. We compared the wet weight with the dry weight and recorded the wet-to-dry ratio.

The lung tissue was washed with PBS, fixed in 4% formalin, and embedded in paraffin. The tissue samples were cut into 5 *μ*m-thick slices for histopathological staining with hematoxylin and eosin (H&E), Masson's trichrome, and Sirius red. The pathological damage and collagen deposition in the lung tissue were observed.

### 2.6. Assessment of Lung Fibrosis

The paraffin-embedded sections were taken for immunohistochemical staining of fibrosis markers (collagen, fibronectin, and *α*-SMA). We observed the expression and localization of the markers in the lung tissues. Moreover, the expression of the fibrosis markers was detected by western blot. The supernatant of alveolar lavage fluid was taken, and the content of hydroxyproline was detected by ELISA kit to evaluate the PF damage.

### 2.7. OS in the Lung

The contents of GSH, MDA, and SOD in the lung tissue were measured by appropriate kits. The lung tissue was ground with the aid of tissue lysate at 4°C and centrifuged at 12000 r/min for 5 min; then, the supernatant was taken for protein quantification, and the indexes related to OS were detected. The content of hydrogen peroxide in the lung tissue was measured by enzyme biosensor, and 8-isoproterenol 2*α* was detected by ELISA (8-iso-PGF2*α*). We evaluated the OS level in the lung tissue.

On this basis, the lung tissue was taken to generate frozen sections, and the ROS level was detected by immunofluorescence to evaluate OS in the lung tissue.

### 2.8. Statistical Analysis

We analyzed the results of Masson's trichome and Sirius red immunohistochemistry staining using Image Pro Plus 6.0 software and conducted statistical analyses using GraphPad Prism 8.0 software. The variations among the groups were assessed by one-way analysis of variance (ANOVA), followed by Dunnett's test. All of the quantitative data are expressed as the mean ± standard deviation (SD), and differences were considered statistically significant and highly significant at *P* < 0.05 (^∗^/#) and *P* < 0.01 (^∗∗^/##), respectively.

## 3. Results

### 3.1. Pathological Changes in the Mouse Lung Tissue

**Figure 1:**
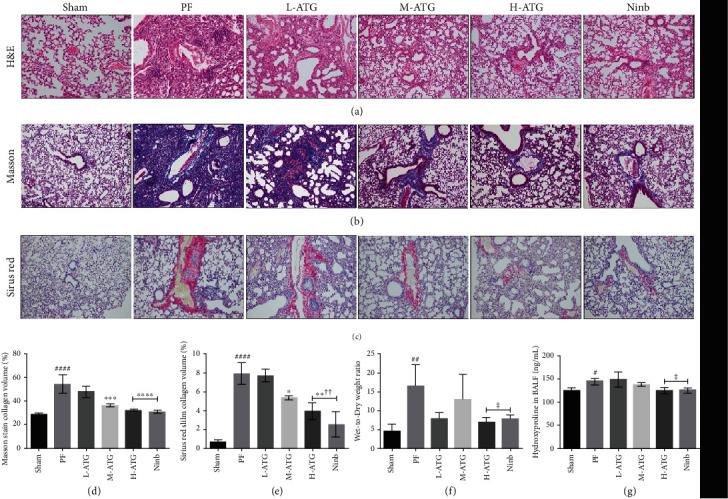
(a) Hematoxylin and eosin staining was used to observe lung injury in mice. (b) Masson's trichrome staining was used to observe pulmonary fibrosis in mice. (c) Sirius red staining was used to observe collagen deposition in the lungs of mice. (d) Collagen distribution volume statistics using Masson's trichrome staining images; and (e) collagen distribution volume statistics for Sirius red staining images (calculated by Image Pro Plus) (^∗^*P* < 0.05, ^∗∗∗^*P*, ^∗∗∗∗^*P* < 0.01 vs. the PF group; ^####^*P* < 0.01 vs. the Sham group). (f, g) The wet-to-dry ratio and hydroxyproline content for each group (^∗^*P* < 0.05 vs. the PF group; ^#^*P* < 0.05, ^##^*P* < 0.01 vs. the Sham group).

The H&E staining results showed that the lung tissue of PF mice had pathological changes, inflammatory infiltration, and destruction of the alveolar tissue structure. The lung tissue of the mice treated with medium- and high-dose ATG was similar to that of the Sham operation group (Figure 1(a)). The results of Masson's trichrome staining showed a large number of collagen fibers in the lung tissue, whereas the lung tissue of the mice in the positive control group was significantly ameliorated. ATG treatment showed an obvious concentration dependence, with the greatest improvement observed in the high-dose group (Figures 1(b) and 1(d)). Sirius red staining showed obvious collagen deposition in the lung tissue of PF mice, and the positive control mice showed a good therapeutic effect. ATG also showed a good therapeutic effect, but it did not show concentration dependence (Figures 1(c) and 1(e)).

We found that the lung wet-to-dry ratio of the PF lung tissue was significantly higher than that of the Sham operation group (Figure 1(f)). We observed no improvement in low- and medium-dose groups, while we observed significant decreases in the high-dose and positive control groups. Data analysis showed differential changes (*P* < 0.05). Next, the mice were pretreated with bronchoalveolar lavage fluid (BALF) (Figure 1(g)). We found that the content of hydroxyproline in BALF increased significantly in mice suffering from PF. We did not observe a significant difference between the low- and medium-dose treatment groups, but we did find a significant difference between the high-dose and the positive control groups. The results showed that high-dose ATG could effectively reduce the secretion of hydroxyproline in mouse BALF.

### 3.2. PF Assessment

**Figure 2:**
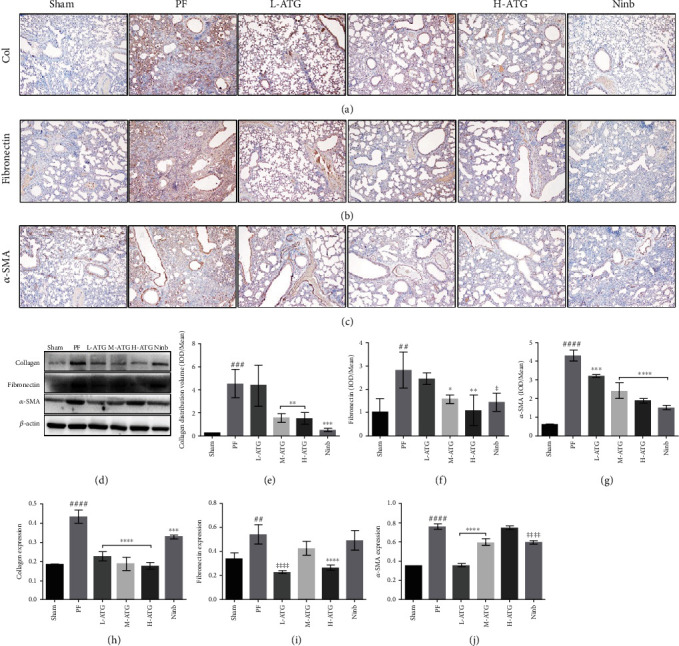
Assessment of pulmonary fibrosis. Immunohistochemical detection of fibrosis biomarkers: (a) collagen type I, (b) fibronectin, and (c) *α*-SMA. (d) Expression of the fibrosis biomarkers by western blot. (e)–(g) Statistical analysis of the fibrosis biomarkers by an immunohistochemical assay (^∗^*P* < 0.05, ^∗∗^*P*, ^∗∗∗^*P*, ^∗∗∗∗^*P* < 0.01 vs. the PF group; ^##^*P*, ^###^*P*, ^####^*P* < 0.01 vs. the Sham group). (h)–(j) Expression of the fibrosis biomarkers by western blot (^∗∗∗^*P*, ^∗∗∗∗^*P* < 0.01 vs. the PF group; ^##^*P*, ^###^*P*, ^####^*P* < 0.01 vs. the Sham group).

Through immunohistochemical analysis of the expression of PF biomarkers (Figures 2(a)–2(c)), we found that the expression levels of collagen, fibronectin, and *α*-SMA in the lung tissue of the PF mice significantly increased, almost over the whole lung tissue, which was consistent with the results of the previous pathological analysis. The positive control group demonstrated a clear therapeutic effect, and the expression of PF markers was effectively inhibited (Figures 2(e)–2(g)). After ATG intervention, we found no obvious therapeutic effect on inhibiting collagen and fibronectin expression in the low-dose group, whereas the medium- and high-dose groups showed better therapeutic effects. In particular, in the high-dose ATG group, the expression of the fibrosis markers in the lungs decreased significantly and tended to be similar to the positive control group.

Next, a western blot was used to verify the expression of the fibrosis biomarkers in the lung tissue (Figure 2(d)). The results showed that ATG effectively inhibited the expression of collagen, while medium-dose ATG and nintedanib showed no inhibitory effect on the fibronectin expression, and high-dose ATG showed no inhibitory effect on the expression of *α*-SMA (Figures 2(h)–2(j)).

### 3.3. OS in the Lungs

**Figure 3:**
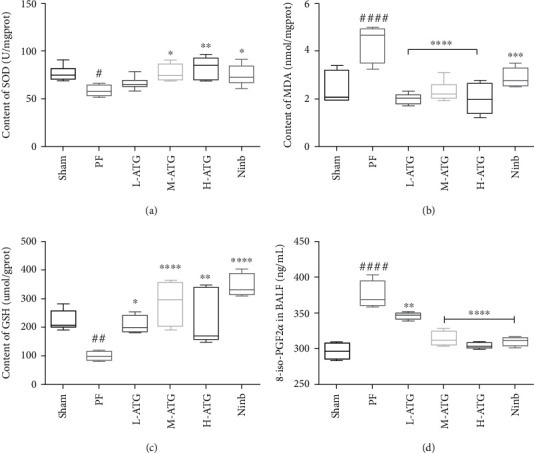
Assessment of oxidative stress in the lungs: (a) SOD, (b) MDA, (c) GSH, and (d) 8-ios-PGF2*α* content in each group (^∗^*P* < 0.05, ^∗∗^*P*, ^∗∗∗^*P*, ^∗∗∗∗^*P* < 0.01 vs. the PF group; ^#^*P* < 0.05, ^##^*P*, ^####^*P* < 0.01 vs. the Sham group).

To determine the level of OS in the lungs, the lung tissue was ground with liquid nitrogen, proteins were extracted, and the contents of SOD, MDA, and GSH were detected (Figures 3(a)–3(c)). We found that the contents of SOD and GSH in the lung tissue of PF mice decreased significantly, whereas the content of MDA increased significantly. ATG intervention effectively improved the OS of the lung tissue. The effects of medium- and high-dose ATG were more stable, whereas the regulation effect of low-dose ATG on SOD was not obvious. Subsequently, we analyzed 8-iso-prostaglandin PGF2*α* (8-iso-PGF2*α*) in the alveolar lavage fluid of the mice (Figure 3(d)). The 8-iso-PGF2*α* content in PF increased significantly, and medium and high doses of ATG effectively inhibited the 8-iso-PGF2*α* expression.

### 3.4. Expression of OS Regulatory Factors in Lungs

**Figure 4:**
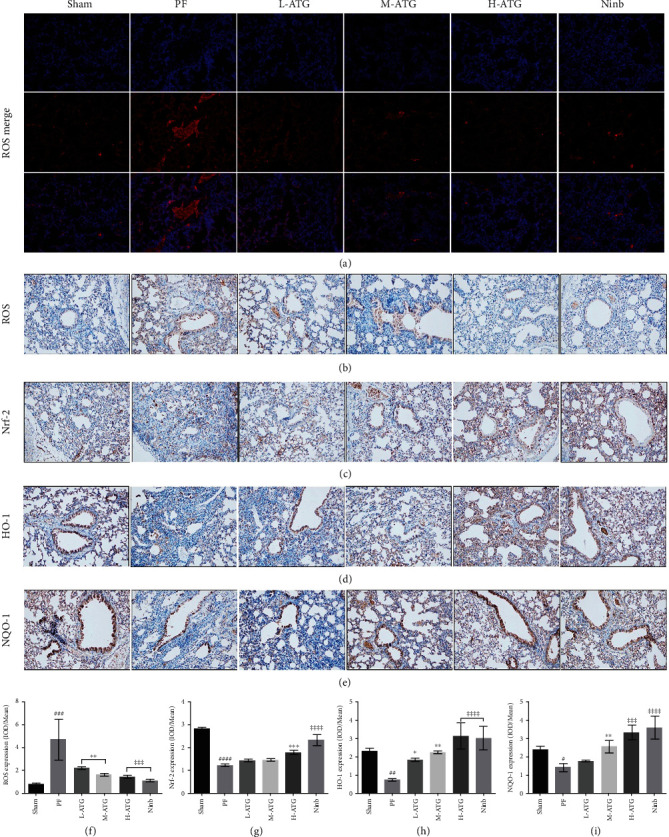
(a) Immunofluorescence detection of ROS. (b)–(e) Expression of ROS, Nrf-2, HO-1, and NQO-1 by immunohistochemistry. (f)–(i) Statistical analysis of ROS, Nrf-2, HO-1, and NQO-1 by immunohistochemical detection (^∗∗^*P*, ^∗∗∗^*P*, ^∗∗∗∗^*P* < 0.01 vs. the PF group; ^#^*P* < 0.05, ^##^*P*, ^###^*P*, ^####^*P* < 0.01 vs. the Sham group).

The expression of ROS in the mouse lungs was detected by immunofluorescence (Figure 4(a)). Using fluorescence microscopy, the expression of ROS in the lung tissue of PF mice was significantly enhanced, which was effectively reduced by ATG; this effect was the most noticeable in the high-dose ATG group, the results of which were similar to those of the nintedanib treatment group. The expression of OS-related regulatory factors was detected by immunohistochemistry and western blot. We examined the OS regulatory factor nuclear factor-related factor 2 (Nrf-2) (Figure 4(b)) and its regulated antioxidant factors, heme oxygenase 1 (HO-1) (Figure 4(c)) and quinone oxidoreductase 1 (NQO1) (Figure 4(d)). We found that high-dose ATG intervention effectively enhanced the expression of these three factors and alleviated the OS pressure in the lung tissue of PF mice (Figures 4(g)–4(i)).

### 3.5. Expression of the TGF-*β*/Akt Signaling Pathway in Mouse Lungs

**Figure 5:**
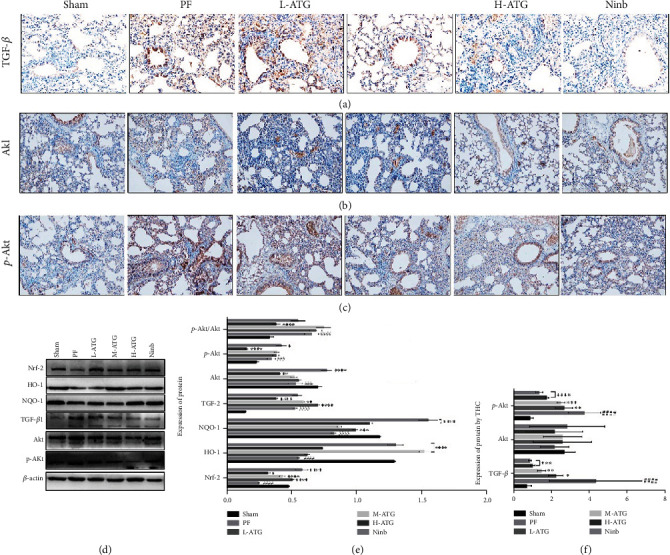
(a)–(c) Immunohistochemistry detection of TGF-*β*, Akt, and p-Akt. (d) Expression of antioxidant factors and TGF-*β*/Akt tested by western blot. (e) Expression of antioxidant factors and TGF-*β*/Akt by western blot (^∗^*P* < 0.05, ^∗∗^*P*, ^∗∗∗^*P*, ^∗∗∗∗^*P* < 0.01 vs. the PF group; ^###^*P*, ^####^*P* < 0.01 vs. the Sham group). (f) Statistical analysis by immunohistochemistry (^∗^*P* < 0.05, ^∗∗^*P*, ^∗∗∗^*P*, ^∗∗∗∗^*P* < 0.01 vs. the PF group; ^####^*P* < 0.01 vs. the Sham group).

The TGF-*β*/Akt signaling pathway was next examined and tested by immunohistochemistry and western blot (Figure 5). We found that the expression of TGF-*β* in the lung tissue of PF mice was significantly enhanced, while ATG effectively inhibited its expression (Figure 5(a)). We also detected the expression of Akt and phosphorylated Akt (p-Akt) (Figures 5(b) and 5(c)), and immunohistochemistry showed that the expression of p-Akt was significantly enhanced under the regulation of TGF-*β* (Figure 5(e)). The western blot displayed similar results (Figures 5(d) and 5(f)). These results suggested that ATG indirectly regulates the TGF/Akt signaling pathway through its antioxidant effect.

## 4. Discussion

In the early stages of PF, various injuries and stimuli can recruit circulating monocytes to differentiate into macrophages after entering the diseased tissue. The differentiation of macrophages can form two extreme phenotypes with different inflammatory states, namely, classically activated M1 macrophages and alternatively activated M2 macrophages. These subgroups have different gene expression profiles and biomarkers. In the early stage of tissue injury, apoptotic cells recruit proinflammatory monocytes to aggregate, differentiate into classically activated M1 macrophages and clear necrotic tissue, and express numerous proinflammatory cytokines, including tumor necrosis factor (TNF-*α*), interleukin-6 (IL-6), and interferon *γ* (IFN-*γ*). Subsequently, the proinflammatory signal is inhibited, favoring the differentiation into alternatively activated M2 macrophages, which express high levels of anti-inflammatory cytokines and growth factors, such as IL-4, IL-10, and transforming growth factor *β* (TGF-*β*) [25, 26]. Cytokines stimulate the differentiation and proliferation of epithelial and endothelial cells, restore tissue morphological structure, and promote the phenotypic transformation of fibroblasts into myofibroblasts to synthesize and secrete ECM and promote tissue repair [27]; however, if M2 macrophages are continuously activated, they may stimulate myofibroblasts to further secrete ECM, which can lead to fibrosis [25].

It has been found that the long-term efficacy of treatment by simply inhibiting inflammation is unsatisfactory. Subsequently, it has been shown that the occurrence of PF is closely related to the oxidation/antioxidant imbalance caused by the accumulation of ROS [28–30]. Compared to healthy individuals, OS biomarkers in the exhaled respiratory condensate of patients with PF contain hydrogen peroxide (H_2_O_2_) and 8-iso-PGF2 *α*. In addition, the contents of Nrf-2 and its regulated antioxidant factors, HO-1 and NQO1, in the BALF of patients with PF are nearly five times higher, while GSH is decreased in the epithelial cell lining fluid and sputum of patients with PF [31, 32].

ROS has an important impact on the development of PF. Persistent lung injury can produce ROS, which can cause apoptosis of ACEs, damage to the basement membrane, a transformation of stroma to the epithelium, destruction of lung structure, and damage of alveolar gas exchange [33]. ROS produced by alveolar type II cell injury can cause an OS response, which can not only induce epithelial cell apoptosis but also activate intracellular signaling pathways and upregulate the synthesis and release of TGF-*β*, finally leading to lung injury and fibrosis. Moreover, the intracellular signaling triggered by OS can stimulate fiber proliferation and the expression of TGF-*β*, thereby accelerating the development of PF [34]. This finding also suggests that PF can be delayed through an antioxidant effect.

In this study, through histopathological staining analysis, we confirmed that ATG effectively inhibited the expression and deposition of collagen fibers in the lungs of PF mice. The immunohistochemistry and western blot results showed that ATG effectively reduced the expression of the examined fibrosis biomarkers. However, the mechanism by which ATG affects PF remains unknown. ATG has been shown to have a good antioxidant effect, and considering the key regulatory role of OS in the occurrence and development of PF, we tested the key indicators of OS in the lungs of PF mice. The results showed that the expression of MDA increased, and the activities of GSH and SOD decreased in the lungs of PF mice, while all indicators were improved after ATG treatment. These findings suggest that ATG can treat PF through its antioxidant effects.

First, we detected the expression of ROS in mouse lung tissue by immunofluorescence. The immunofluorescence results showed that the content of ROS in mouse lung tissue increased significantly after fibrosis, confirming previous findings that the development of PF is closely related to OS. There was no obvious expression of ROS in the lung tissue of ATG-treated mice, which also showed that ATG had an antioxidant effect. Although these findings demonstrate that ATG can inhibit the process of bleomycin-induced PF through antioxidant effects, the specific details of the effect remain unclear.

Nrf2 is a central regulator of intracellular redox homeostasis and an important transcription factor involved in regulating the cellular OS response. By inducing and regulating the constitutive and inducible expression of a series of antioxidant proteins, Nrf2 can reduce the cell damage caused by ROS and electrophiles, maintain the cells in a stable state, and maintain the dynamic redox balance [35, 36]. HO-1 and NQO-1, regulated by Nrf-2, have antioxidant, anti-inflammatory, and antiapoptotic effects and are involved in signal transduction, immune regulation, and inhibition of the adhesion molecule expression. The strong adaptive response of HO-1 to various stimulating factors suggests that HO-1 plays an important role in preventing the inflammatory process and oxidative tissue damage [37].

Second, considering that Nrf-2, HO-1, and NQO-1 play important roles, we immunohistochemically detected their expression levels. We found that high-dose ATG intervention effectively enhanced the expression of these three factors and alleviated the OS pressure in the lung tissue of PF mice. We next detected the regulatory factors by western blot. These results showed that medium and high doses of ATG were able to promote the expression of OS-related factors, effectively improve the expression of antioxidant substances in the lung tissue, and strengthen the antioxidant effect.

Although we proved experimentally that ATG can inhibit PF through antioxidative effects, previous studies have reported that TGF-*β* is the most direct regulator of fibrosis, and that regulation of TGF-*β* could effectively reduce collagen secretion. Finally, to clarify how ATG regulates TGF-*β*, we analyzed TGF-*β* by immunohistochemistry and western blot. We found that the expression of TGF-*β* in the lung tissue of PF mice was significantly enhanced, which was effectively inhibited by ATG. We also detected the expression of Akt and phosphorylated Akt (p-Akt) and found that the expression of p-Akt was significantly enhanced under the regulation of TGF-*β*. This result suggested that ATG indirectly regulates the TGF/Akt signaling pathway through its antioxidant effects.

## Data Availability

The data were all included in the manuscript.

## References

[1] Richeldi L., Collard H. R., Jones M. G. (2017). Idiopathic pulmonary fibrosis. *Lancet*.

[2] Wynn T. A. (2008). Cellular and molecular mechanisms of fibrosis. *Journal of Pathology*.

[3] Wynn T. A., Ramalingam T. R. (2012). Mechanisms of fibrosis: therapeutic translation for fibrotic disease. *Nature Medicine*.

[4] Hutchinson J., Fogarty A., Hubbard R., McKeever T. (2015). Global incidence and mortality of idiopathic pulmonary fibrosis: a systematic review. *European Respiratory Journal*.

[5] Rogliani P., Calzetta L., Cavalli F., Matera M. G., Cazzola M. (2016). Pirfenidone, nintedanib and _N_ -acetylcysteine for the treatment of idiopathic pulmonary fibrosis: A systematic review and meta-analysis. *Pulmonary Pharmacology & Therapeutics*.

[6] Flaherty K. R., Wells A. U., Cottin V. (2019). intedanib in progressive fibrosing interstitial lung diseases. *The New England Journal of Medicine*.

[7] Wu H., Yu Y., Huang H. (2020). Progressive pulmonary fibrosis is caused by elevated mechanical tension on alveolar stem cells. *Cell*.

[8] Canestaro W. J., Forrester S., Ho L., Devine B. (2015). Drug therapy for treatment of idiopathic pulmonary fibrosis: a systematic review and network meta-analysis. *Chest*.

[9] Psathakis K., Mermigkis G., Papatheodorou S. (2006). Exhaled markers of oxidative stress in idiopathic pulmonary fibrosis. *European Journal of Clinical Investigation*.

[10] Lu M., Ji J., Jiang Z., You Q. D. (2016). The Keap1-Nrf2-ARE pathway as a potential preventive and therapeutic target: an update. *Medicinal Research Reviews*.

[11] Cantin A. M., North S. L., Fells G. A., Hubbard R. C., Crystal R. G. (1987). Oxidant-mediated epithelial cell injury in idiopathic pulmonary fibrosis. *The Journal of Clinical Investigation*.

[12] Cicko S., Grimm M., Ayata K. (2015). Uridine supplementation exerts anti- inflammatory and anti-fibrotic effects in an animal model of pulmonary fibrosis. *Respiratory Research*.

[13] Chen C., Yang S., Zhang M. (2016). Triptolide mitigates radiation-induced pulmonary fibrosis via inhibition of axis of alveolar macrophages-NOXes-ROS-myofibroblasts. *Cancer Biology & Therapy*.

[14] Karampitsakos T., Woolard T., Bouros D., Tzouvelekis A. (2017). Toll-like receptors in the pathogenesis of pulmonary fibrosis. *European Journal of Pharmacology*.

[15] Ge A., Ma Y., Liu Y. N. (2016). Diosmetin prevents TGF-*β*1-induced epithelial-mesenchymal transition via ROS/MAPK signaling pathways. *Life Sciences*.

[16] Cameli P., Carleo A., Bergantini L., Landi C., Prasse A., Bargagli E. (2020). Oxidant/antioxidant disequilibrium in idiopathic pulmonary fibrosis pathogenesis. *Inflammation*.

[17] Kato K., Hecker L. (2020). NADPH oxidases: pathophysiology and therapeutic potential in age-associated pulmonary fibrosis. *Redox Biology*.

[18] Gao Q., Yang M., Zuo Z. (2018). Overview of the anti-inflammatory effects, pharmacokinetic properties and clinical efficacies of arctigenin and arctiin from _Arctium lappa_ L. *Acta Pharmacologica Sinica*.

[19] He Y., Fan Q., Cai T. (2018). Molecular mechanisms of the action of Arctigenin in cancer. *Biomedicine & Pharmacotherapy*.

[20] Zhang Y., Yang Y. (2018). Arctigenin exerts protective effects against myocardial infarction via regulation of iNOS, COX-2, ERK1/2 and HO-1 in rats. *Molecular Medicine Reports*.

[21] du Sert N. P., Hurst V., Ahluwalia A. (2020). The ARRIVE guidelines 2.0: updated guidelines for reporting animal research. *PLOS Biology*.

[22] Wang L., Zhang P., Li X., Zhang Y., Zhan Q., Wang C. (2019). Low-molecular-weight fucoidan attenuates bleomycin-induced pulmonary fibrosis: possible role in inhibiting TGF-*β*1-induced epithelial-mesenchymal transition through ERK pathway. *American Journal of Translational Research*.

[23] Wu N., Li Z., Wang J. (2021). Low molecular weight fucoidan attenuating pulmonary fibrosis by relieving inflammatory reaction and progression of epithelial-mesenchymal transition. *Carbohydrate Polymers*.

[24] Dong H. D., Xue T., Liu Y. J. (2022). Low molecular weight fucoidan inhibits pulmonary fibrosis in vivo and in vitro via antioxidant activity. *Oxidative Medicine and Cellular Longevity*.

[25] Cheng P., Li S., Chen H. (2021). Macrophages in lung injury, repair, and fibrosis. *Cell*.

[26] Rao L., Wang Y., Zhang L. (2021). IL-24 deficiency protects mice against bleomycin-induced pulmonary fibrosis by repressing IL-4-induced M2 program in macrophages. *Cell Death & Differentiation*.

[27] Wang Y., Zhang L., Wu G. R. (2021). MBD2 serves as a viable target against pulmonary fibrosis by inhibiting macrophage M2 program. *Science Advances*.

[28] Cui Y., Xin H., Tao Y., Mei L., Wang Z. (2021). Arenaria kansuensis attenuates pulmonary fibrosis in mice via the activation of Nrf2 pathway and the inhibition ofNF‐kB/TGF‐beta1/Smad2/3 pathway. *Phytotherapy Research*.

[29] Tao N., Li K., Liu J., Fan G., Sun T. (2021). Liproxstatin-1 alleviates bleomycin-induced alveolar epithelial cells injury and mice pulmonary fibrosis via attenuating inflammation, reshaping redox equilibrium, and suppressing ROS/p53/*α*-SMA pathway. *Biochemical and Biophysical Research Communications*.

[30] Sugizaki T., Tanaka K., Asano T. (2019). Idebenone has preventative and therapeutic effects on pulmonary fibrosis via preferential suppression of fibroblast activity. *Cell Death Discovery*.

[31] Forman H. J., Zhang H. (2021). Targeting oxidative stress in disease: promise and limitations of antioxidant therapy. *Nature Reviews Drug Discovery*.

[32] Liu R. M., Vayalil P. K., Ballinger C. (2012). Transforming growth factor *β* suppresses glutamate-cysteine ligase gene expression and induces oxidative stress in a lung fibrosis model. *Free Radical Biology & Medicine*.

[33] Xiong M., Zhao Y., Mo H., Yang H., Yue F., Hu K. (2021). Intermittent hypoxia increases ROS/HIF-1*α* 'related oxidative stress and inflammation and worsens bleomycin-induced pulmonary fibrosis in adult male C57BL/6J mice. *International Immunopharmacology*.

[34] Sharma A., Tewari D., Nabavi S. F., Nabavi S. M., Habtemariam S. (2021). Reactive oxygen species modulators in pulmonary medicine. *Current Opinion in Pharmacology*.

[35] Zhang Z., Qu J., Zheng C. (2018). Nrf2 antioxidant pathway suppresses Numb-mediated epithelial–mesenchymal transition during pulmonary fibrosis. *Cell Death & Disease*.

[36] Zhou W., Mo X., Cui W. (2016). Nrf2 inhibits epithelial-mesenchymal transition by suppressing snail expression during pulmonary fibrosis. *Scientific Reports*.

[37] Huai B., Ding J. (2020). Atractylenolide III attenuates bleomycin-induced experimental pulmonary fibrosis and oxidative stress in rat model via Nrf2/NQO1/HO-1 pathway activation. *Immunopharmacology and Immunotoxicology*.

